# Evaluation of the Immunogenic Response of a Novel Enterobactin Conjugate Vaccine in Chickens for the Production of Enterobactin-Specific Egg Yolk Antibodies

**DOI:** 10.3389/fimmu.2021.629480

**Published:** 2021-04-02

**Authors:** Ximin Zeng, Huiwen Wang, Canghai Huang, Catherine M. Logue, Nicolle L. Barbieri, Lisa K. Nolan, Jun Lin

**Affiliations:** ^1^ Department of Animal Science, The University of Tennessee, Knoxville, TN, United States; ^2^ College of Fisheries, Jimei University, Xiamen, China; ^3^ College of Veterinary Medicine, University of Georgia, Athens, GA, United States

**Keywords:** enterobactin conjugate vaccine, chicken, egg yolk antibody, Gram-negative pathogen, nutritional immunity

## Abstract

Passive immunization with specific egg yolk antibodies (immunoglobulin Y, IgY) is emerging as a promising alternative to antibiotics to control bacterial infections. Recently, we developed a novel conjugate vaccine that could trigger a strong immune response in rabbits directed against enterobactin (Ent), a highly conserved siderophore molecule utilized by different Gram-negative pathogens. However, induction of Ent-specific antibodies appeared to be affected by the choice of animal host and vaccination regimen. It is still unknown if the Ent conjugate vaccine can trigger a specific immune response in layers for the purpose of production of anti-Ent egg yolk IgY. In this study, three chicken vaccination trials with different regimens were performed to determine conditions for efficient production of anti-Ent egg yolk IgY. Purified Ent was conjugated to three carrier proteins, keyhole limpet hemocyanin (KLH), bovine serum albumin (BSA) and CmeC (a subunit vaccine candidate), respectively. Intramuscular immunization of Barred Rock layers with KLH-Ent conjugate four times induced strong immune response against whole conjugate vaccine but the titer of Ent-specific IgY did not change in yolk with only a 4 fold increase detected in serum. In the second trial, three different Ent conjugate vaccines were evaluated in Rhode Island Red pullets with four subcutaneous injections. The KLH-Ent or CmeC-Ent conjugate consistently induced high level of Ent-specific IgY in both serum (up to 2,048 fold) and yolk (up to 1,024 fold) in each individual chicken. However, the Ent-specific immune response was only temporarily and moderately induced using a BSA-Ent vaccination. In the third trial, ten White Leghorn layers were subcutaneously immunized three times with KLH-Ent, leading to consistent and strong immune response against both whole conjugate and the Ent molecule in each chicken; the mean titer of Ent-specific IgY increased approximately 32 and 256 fold in serum and yolk, respectively. Consistent with its potent binding to various Ent derivatives, the Ent-specific egg yolk IgY also inhibited *in vitro* growth of a representative *Escherichia coli* strain. Together, this study demonstrated that the novel Ent conjugate vaccine could induce strong, specific, and robust immune response in chickens. The Ent-specific hyperimmune egg yolk IgY has potential for passive immune intervention against Gram-negative infections.

## Introduction

Enteric foodborne pathogens are a prominent public health challenge in the USA for both food animals and humans. Despite extensive efforts for developing approaches for enteric pathogen control in food animals, the incidence of bacterial infectious diseases caused by enteric pathogens remains high and has resulted in significant economic loss, such as colibacillosis and salmonellosis in poultry industry (1). In addition, the use of antibiotics in animal feed for the purpose of growth performance improvement, prophylaxis, or disease treatment has led to increasing antibiotic resistance in various zoonotic bacterial pathogens (2). Due to the concerns of antibiotic resistance development and its threat to public health (3), development of alternatives to antibiotics, such as vaccines, is highly desired (4).

Recently, discovery of effective vaccines against bacterial pathogens has encountered some bottlenecks (5). One important reason is that bacterial vaccines often target large surface-exposed components that are usually redundant or versatile, enabling bacteria to evade the host immune response (6). Therefore, new strategies targeting extracellular essential nutritional components, which are often small and highly conserved molecules, has been recently proposed (also termed as nutritional immunity) (7, 8). Iron is an essential nutrient for bacterial survival and colonization in iron-restricted host environment, such as the gastrointestinal tract. To compete for iron, many members of Enterobacteriaceae family, such as *Escherichia coli* and *Salmonella* spp., can synthesize enterobactin (Ent) and/or associated derivatives (*e.g.* salmochelins), the powerful catecholate siderophores displaying extremely high affinity to ferric iron. During evolution, host acute phase protein lipocalin, due to its potent binding ability to Ent, could counteract Ent-mediated iron acquisition and serve as a bacteriostatic innate immune effector against enteric bacterial pathogens (9). Recently, novel Ent conjugate vaccines have also been successfully developed to induce lipocalin-like Ent-specific antibodies in the host, which showed significant potential for broad applications to prevent and control various Gram-negative infections in humans and animals (10, 11). In particular, we have developed an efficient method to prepare a new type of Ent conjugate vaccine that can induce high level of Ent-specific antibodies in rabbits (up to 4,096 fold increase) (10, 12). More importantly, the Ent-specific antibodies have been demonstrated to function similarly as lipocalin to interfere with Ent-dependent growth of different Gram-negative pathogens (10, 12). Notably, induction of Ent-specific antibodies appeared to be affected by the choice of animal hosts and vaccination regimens because another reported Ent conjugate vaccine only induced weak Ent-specific immune response (< 4 fold increase) in the immunized mice (11). Given substantial differences in the genetic and immune systems in animal hosts (13, 14), assessment of immunogenicity of the new Ent conjugate vaccine in different and important animal hosts, such as chickens, is highly desirable.

In addition to its critical role in human food security and sustainability, chicken also can produce unique egg yolk antibodies (immunoglobulin Y, IgY) used for passive immune intervention (15). Production of egg yolk antibodies is characterized as high yield, low cost, and good animal welfare (less invasive collection process). The amount of egg yolk antibodies produced yearly by an immunized hen is over 22,500 mg with up to 10% being antigen-specific antibodies (16, 17). In addition, antibodies in eggs are compartmented very well with IgY enriched in the yolk (18, 19) while IgA and IgM are primarily distributed in the egg white (20), which facilitates purification of large quantities of desired IgY at low cost if needed (21). Therefore, immunization of layer hens with novel vaccines, such as our recently developed Ent conjugates (10), may lead to better production of hyperimmune egg yolk antibodies for passive prophylactic and therapeutic treatment against Gram-negative infections in food animals and humans (22).

In this study, we explored the feasibility of using layers as an efficient, specific and sustainable bio-reactor to produce large amount of Ent-specific hyperimmune egg yolk antibodies. Different Ent conjugates, layer breeds and immunization routes were examined. The keyhole limpet hemocyanin-Ent (KLH-Ent) conjugate vaccine was demonstrated to consistently induce stable and high titer of Ent-specific egg yolk antibodies *via* subcutaneous route in at least two layer breeds. We also showed the anti-Ent egg yolk antibodies could bind a variety of Ent derivatives including salmochelins and significantly inhibited the growth of *E. coli* MG1655 in an *in vitro* growth assay. These findings have laid a solid foundation for us to evaluate the prophylactic and therapeutic efficacy of the Ent-specific egg yolk antibodies for mitigation of Gram-negative infections in food animals and even humans in the future.

## Materials and Methods

### Preparation of Ent Conjugate

An Ent transport mutant of *E. coli* AN102 (JL122) was used for Ent purification as described previously (23, 24). The purified Ent was coupled with BSA (bovine serum albumin, Cat No. PI77110, Fisher Scientific), KLH (Cat No. PI77600, Fisher Scientific) and recombinant CmeC (25), respectively, by following the method detailed in our previous publication (10). Each batch of BSA-Ent, KLH-Ent and CmeC-Ent conjugates were subjected to SDS-PAGE analysis to confirm the success of conjugation. Finally, the Ent conjugates (~1 mg/ml in PBS) were stored at -20°C prior to use.

### SDS-PAGE and Immunoblotting

The carrier proteins and corresponding conjugation reaction mixes were subjected to SDS-PAGE analysis (12% [w/v] polyacrylamide gel) and stained with Coomassie blue R-250. The dot blotting and Western immunoblotting were performed as described previously (10, 26, 27). For dot blotting, approximately 1 µl of conjugate sample (1 mg/ml) was spotted onto nitrocellulose membranes, air dried and blocked with blocking buffer (PBS containing 0.05% of Tween 20 and 5% of skim milk) at room temperature (22°C) for 1 hour. Subsequently, the nitrocellulose membranes were incubated at room temperature with the rabbit anti-KLH-Ent antiserum (10) that was 1:200 diluted in blocking buffer for 1 hour. After washing four times with PBS-T solution (PBS containing 0.05% Tween 20), the nitrocellulose membranes were incubated with goat anti-rabbit IgG-horseradish peroxidase secondary antibody (SeraCare, Milford, MA, 1:2,000 diluted in blocking buffer) at room temperature for 1 hour. After the membranes were washed four times with PBS-T solution, the membranes were then developed with the 4CN Membrane Peroxidase Substrate System (KPL, Gaithersburg, MD). For Western immunoblotting, the procedure was same as above for dot blotting except that the membrane was blocked with blocking buffer at 4°C overnight.

### Immunization of Layers With Ent Conjugate Vaccines

**Figure 1:**
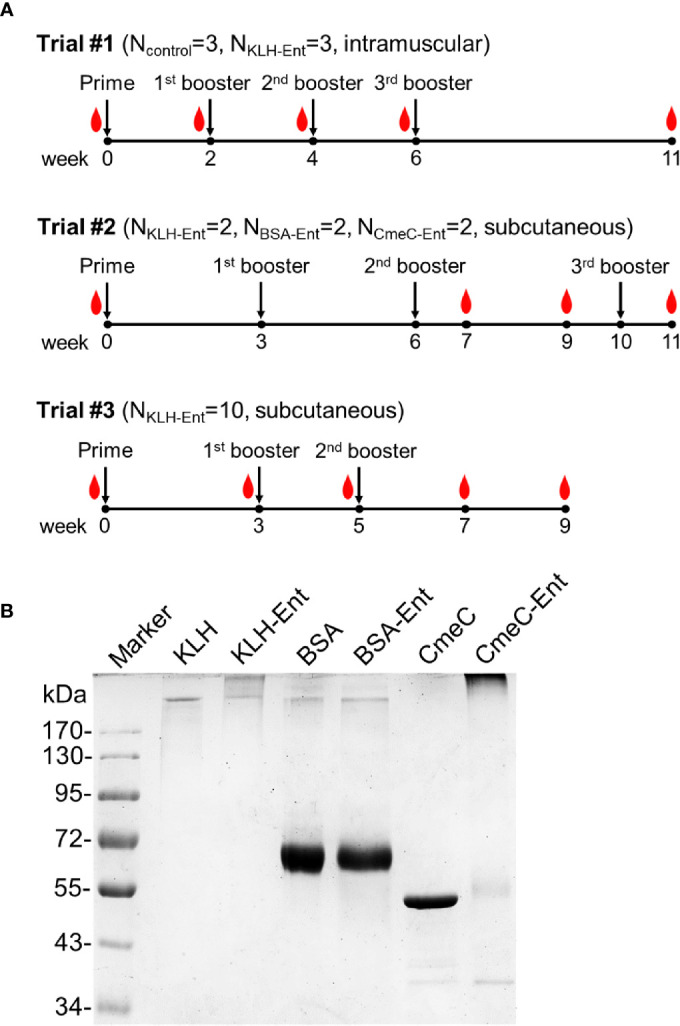
Experimental design for evaluating immunogenicity of the new Ent conjugate vaccines in chickens for production of Ent-specific egg yolk antibodies. **(A)** Diagram of three immunization trials. The prime and booster immunizations are indicated by downwards arrows while blood sampling was denoted as blood drop. The conjugate vaccines, number of animals in each group and the administration routes are described within the parentheses after each specific trial number. Eggs were collected throughout the trials. **(B)** SDS-PAGE analysis of three Ent conjugates. Different carrier proteins (*i.e.*, KLH, BSA, and CmeC) and corresponding conjugates are indicated above image. The far left lane is the EZ-Run™ prestained Rec protein ladder (Fisher Scientific) with molecular weight (kDa) indicated.

Three independent immunization trials were performed (**Figure 1A**). The chicken experiments were approved by Institutional Animal Care and Use Committee at The University of Tennessee (IACUC No. 1387, Trial #1), Pacific Immunology Corp. (Ramona, CA) (Trial #2), and University of Georgia (IACUC No. A2018 01-014-Y1-A0, Trial #3) prior to the start of corresponding animal experiments.

In the first trial (Trial #1), six Barred Rock pullets were purchased from Murray McMurray Hatchery (Webster City, IA) and raised in individual cages. When all the pullets started to consistently lay eggs, three layers received primary immunization *via* intramuscular injection (in pectoral muscles) with 100 µg of KLH-Ent conjugate emulsified with Freund’s complete adjuvant, followed by three booster immunizations (100 µg of KLH-Ent conjugate emulsified with Freund’s incomplete adjuvant) every other week (**Figure 1A**, top panel). The other three layers did not receive vaccination and served as non-immunized control. Notably, although Freund’s complete adjuvant is not allowed in the practice of livestock production, both Freund’s complete and incomplete adjuvants are approved and commonly used in different laboratory animals, including chickens. Blood samples were collected from the wing vein of each chicken immediately prior to each immunization and upon termination (**Figure 1A**, top panel) to monitor circulating IgY antibodies. Eggs were collected every day from each chicken and stored at 4°C prior to egg yolk processing and analysis of specific IgY level.

The second trial (Trial #2) was performed in a professional animal immunization company (Pacific Immunology, Ramona, CA). Briefly, six Rhode Island Red layers were assigned to three immunization groups. Layers in each group received primary immunization *via* subcutaneous injection (under the neck skin near the shoulders) with 100 µg of BSA-Ent, KLH-Ent, or CmeC-KLH conjugates (2 birds per conjugate), respectively, which were emulsified with Freund’s complete adjuvant prior to injection. Subsequently, the layers received three booster immunizations (100 µg of Ent conjugate emulsified with Freund’s incomplete adjuvant) *via* subcutaneous route every 3-4 weeks. Blood samples were collected from the wing vein of each chicken at different time points (as indicated in **Figure 1A**, middle panel) to monitor circulating IgY. The eggs were collected throughout whole trial. Egg yolks were separated, pooled between each immunization, and sent together with each batch of blood samples to PI at the University of Tennessee *via* overnight express. Upon arrival, all samples were stored at 4°C prior to analysis.

In the third trial (Trial #3), fertilized SPF White Leghorn eggs were purchased from Charles River Laboratories (Wilmington, MA) and hatched in the Poultry Diagnostic and Research Center at the University of Georgia. Birds were housed individually from 18 weeks of age prior to lay. At age of approximately 26 weeks and in full lay, ten layers were immunized subcutaneously in the neck with 100 µg of KLH-Ent conjugate that was emulsified with Freund’s complete adjuvant. Two booster immunizations (100 µg of Ent conjugate emulsified with Freund’s incomplete adjuvant) were performed at 3 and 5 weeks after the primary immunization, respectively. Blood samples were collected from the wing vein of each chicken at different time points (as indicated in **Figure 1A**, bottom panel) to monitor circulating IgY levels. Eggs were collected every day from each chicken and stored at 4°C prior to egg yolk processing and analysis.

### Enzyme-Linked Immunosorbent Assay (ELISA) – IgY Titer Evaluation

The specific IgY titers in serum and egg yolk samples were measured by indirect ELISA, which was performed by following published protocols (10, 25) with slight modifications. Microtiter plates (Nunc-MaxiSorp™ Plate, Thermo Fisher scientific) were coated with 100 μL of appropriate antigens (detailed below) in coating buffer (sodium bicarbonate/carbonate buffer, pH 9.6) overnight at room temperature. Specifically, to determine specific immune response to the whole conjugate, the corresponding Ent conjugate (30 ng/well) was used to coat ELISA plates. To determine the level of specific IgY directed against small molecule Ent, BSA-Ent (2 µg/well) was used as coating antigens for the samples from the layers immunized with KLH-Ent or CmeC-Ent while KLH-Ent (2 µg/well) was used as coating antigen for the samples from the layers immunized with BSA-Ent conjugate. The coated plates were blocked with either 5% skim milk (for KLH-Ent and CmeC-Ent immunized group) or 1% gelatin (for BSA-Ent immunized group) in PBS-T solution for 1 hour at room temperature; using gelatin blocking buffer is to eliminate the potential interference effect caused by the BSA in skim milk during examining immune response to the BSA-Ent conjugate. Subsequently, chicken serum and egg yolk samples were 2-fold serially diluted in blocking solution and incubated with the corresponding coated plates for 1 hour at room temperature. Following washing 4 times with PBS-T solution, peroxidase-conjugated goat anti-chicken-IgY secondary antibody was added (1:1000 dilution in blocking solution) and the plates were incubated at room temperature for 1 hour, followed by washing with PBS-T solution four times. The plates were finally developed using an ABTS [2,2’-azinobis (3-ethylbenzthiazolinesulfonic acid)] peroxidase substrate (KPL); the reaction was stopped at 30 min by adding 100 µL of stop solution (1% SDS). The absorbance was measured at an optical density of 405 nm using an ELX808 spectrometer (BioTek Instruments, Winooski, VT) and data were collected using Gen5 software (version 2.03.1; BioTek Instruments). The wells in microtiter plates without addition of primary antibody were served as the background control (blank). Endpoint titer was defined as the last dilution at which optical density (OD_405nm_) of sample wells exceeded the cutoff value (mean OD_405nm_ plus 3 × standard deviation of pre-immune sera at 512-fold dilution). Titer was expressed as the log_2_ of reciprocal of the end point dilution. Duplicate measurements were performed for each sample.

### ELISA – Assess Anti-Ent Egg yolk IgY Binding to Ent Derivatives

The capability of anti-Ent egg yolk antibodies from the KLH-Ent immunized chickens to bind various Ent derivatives was determined by using a recently developed ELISA method (10) with modifications. We first synthesized and purified several major Ent derivatives that were directly used as coating antigens for the ELISA. The ferric Ent complex was prepared by mixing Ent with a 1.2 equivalent of FeCl_3_ as described before (28). Linearized Ent, 2,3-dihydroxybenzoylserine trimer (DHBS)_3_, was produced by hydrolysis of Ent by recombinant IroE, followed by HPLC purification as described in our previous publication (28). Salmochelins (monoglucosylated Ent [Sal-1], diglucosylated Ent [Sal-2], and triglucosylated Ent [Sal-3]) were synthesized by Ent glucosylation with recombinant IroB and purified using HPLC (29, 30). Briefly, 500 μL of the reaction mixture contained 75 mM Tris buffer (pH 7.5), 5 mM MgCl_2_, 2.5 mM TCEP-HCl, 3 mM UPD-Glc, 500 μM Ent, and 1 mM C-glycosyltransferase IroB (29). At different time points, the reaction was stopped by adding 0.1% trifluoroacetic acid (TFA) and analyzed by HPLC using a gradient of 0 to 43.75% CH_3_CN in 0.1% TFA-water over 10 min. Fractions of Sal-1, Sal-2, and Sal-3 were collected, lyophilized, and stored at -20°C prior to use.

To perform the ELISA, Ent and its derivatives were diluted in coating buffer (bicarbonate/carbonate buffer, pH 9.6) to the final concentration of 0.1 mM while BSA-Ent was diluted to 20 μg/ml in the same coating buffer. The microtiter plate (Greiner Microlon, Cat. No. 655001) was coated with each compound (100 μL/well) overnight at room temperature. Subsequently, the ELISA plate with directly coated compounds were subjected to standard ELISA using the same procedure as described above. The pre-immune and post-immune egg yolk samples from KLH-Ent immunized chickens (Trial #3) were diluted 128 fold and served as primary antibody for ELISA analysis.

### Purification of Human Lipocalin-2

His-tagged recombinant human lipocalin-2 was purified from JL1002 (a generous gift from Dr. Charles M. Dozois) (31), an Ent deficient strain (Δ*entD*) ensuring the purified recombinant lipocalin-2 was not bound by endogenous Ent. Purification procedure was described in a previous publication (12). Briefly, JL1002 was induced in the presence of 0.5 mM isopropyl β-d-thiogalactopyranoside for 3 hours. Cells were pelleted, suspended in lysis buffer (50 mM sodium phosphate, 300 mM NaCl, 10 mM imidazole, 5 mM ATP, 5 mM MgCl_2_, 1 mg/ml lysozyme, and 10 mM β-mercaptoethanol, pH 7.0), and incubated on ice for 1 hour. After sonication and centrifugation, the soluble fraction from the lysate was mixed with pre-equilibrated Ni-NTA agarose (Thermo Fisher Scientific) and packed onto a column. Then the column was washed with washing buffer (50 mM sodium phosphate, 300 mM NaCl, 10 mM imidazole, 10% glycerol, 5mM ATP, 5mM MgCl_2_, and 5 mM β-mercaptoethanol, pH 7.0). The recombinant lipocalin-2 was eluted from the column with elution buffer (50 mM sodium phosphate, 300 mM NaCl, 300 mM imidazole, 10% glycerol, and 5 mM β-mercaptoethanol). The eluted recombinant lipocalin-2 was dialyzed against PBS, sterilized by membrane filtration (0.22-μm-pore-size filter), aliquoted, and stored at -20°C. Concentration of the purified human lipocalin-2 was determined using a BCA assay kit (Thermo Fisher Scientific).

### *In Vitro* Growth Assay

The effect of Ent-specific egg yolk antibodies on *E. coli* growth was evaluated using a similar microtiter plate growth assay described in previous publications (10, 12). *E. coli* strain MG1655, known to synthesize Ent only (32), was used as a representative strain in this study. The pre-immune chicken egg yolk served as negative control while recombinant human lipocalin-2 as positive control. Both pre- and post-immune egg yolks from chickens immunized with KLH-Ent (Trial #3) were dialyzed against PBS, and then pasteurized at 56°C for 30 minutes. The *E. coli* strain was inoculated into LB broth supplemented with 0.6 mM 2,2’-dipyridyl. The log-phase cells were washed twice with sterile PBS and subsequently diluted with RPMI medium (Gibco, Dublin, Ireland) to approximately 5 × 10^5^ CFU/mL. In each microplate well, 150 μL of RPMI medium was mixed with 50 μL of pre-immune egg yolk, post-immune egg yolk, or pre-immune egg yolk plus human lipocalin-2 at a final concentration of 37 µg/ml. Subsequently, 5 μL of bacterial suspension (5 × 10^5^ CFU/mL) was added to each well. The microplate was incubated at 37°C for up to 22 hours. At different time points post-inoculation, 20 μL of culture was taken from each well, serially diluted in ice-cold RPMI medium, and plated onto LB agar plates for bacterial enumeration. The colonies were counted after 16 hours of incubation at 37°C for calculating colony forming units (CFUs). The result was expressed as log_10_ CFU/mL. Each treatment was performed in duplicate.

### Statistical Analysis

Repeated measures ANOVA was conducted to compare specific IgY titers in serum and egg yolk. Student’s *t-*test with two-tailed distribution and unpaired sample was conducted for pairwise comparison of *in vitro* growth assay. Data were presented as mean ± standard deviation. A probability level of *P* < 0.05 was considered as a statistically significant difference.

## Results

### Production of Different Ent Conjugate Vaccines.

As shown in **Figure 1B**, both commercial immunogenic carrier proteins (BSA and KLH) and the purified recombinant CmeC, a promising subunit vaccine candidate against *Campylobacter* (25), were successfully conjugated with Ent. Successful conjugation of a carrier protein with Ent is reflected by either slight up shift of the protein band (*e.g.* BSA-Ent) or the smear bands with higher molecular mass (*e.g.* KLH-Ent and CmeC-Ent) when compared with specific unconjugated carrier protein (**Figure 1B**). Furthermore, successful conjugation of Ent to CmeC and BSA was confirmed by dot blotting and Western blotting using the Ent-specific rabbit antiserum generated in our previous study (10) (data not shown).

### Immunization With KLH-Ent *via* Intramuscular Route Only Led to Moderate Increase in Ent-Specific IgY Titer in Serum (Trial #1)

**Figure 2:**
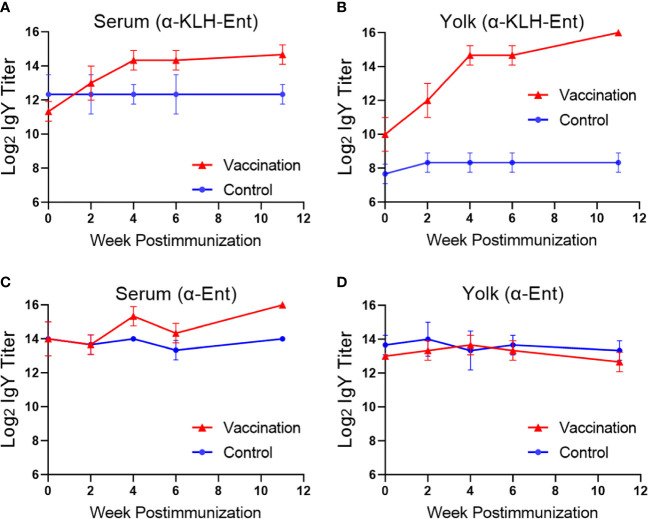
Immune response of Barred Rock layers upon intramuscular immunization with the KLH-Ent conjugate (Trial #1). **(A)** Serum IgY titer against the KLH-Ent conjugate. **(B)** Egg yolk IgY titer against the KLH-Ent conjugate. **(C)** Serum IgY titer against Ent molecule. **(D)** Egg yolk IgY titer against Ent molecule. The chickens without receiving any immunizations served as negative control. Each point represents mean IgY titer ± standard deviation from three chickens with duplicate measurements within each chicken.

In this initial trial, intramuscular immunization of Barred Rock hens with one prime and three booster injections with KLH-Ent was performed (**Figure 1A**, top panel). As shown in **Figure 2**, the KLH-Ent conjugate triggered a strong immune response against the whole conjugate with specific IgY titers increased significantly (*P*<0.05) in serum (approximately 8 fold, **Figure 2A**) and more drastically in yolk (approximately 128 fold, **Figure 2B**) at 11 weeks postimmunization in immunized hens when compared to pre-immune samples. The mean titer of specific IgY directed against the small molecule Ent in serum was moderately elevated but significantly increased at 11 weeks postimmunization in the vaccination group (4 fold, *P* < 0.05) when compared to pre-immune samples (**Figure 2C**). However, the Ent-specific IgY titer did not increase in egg yolks throughout the trial (**Figure 2D**). As expected, in control group, the IgY titers directed against both KLH-Ent and Ent molecule were not significantly changed (**Figure 2**). In this trial, significant variations in terms of specific immune response were observed in individual chickens (**Figure S1**).

### Immunization With KLH-Ent or CmeC-Ent Conjugate *via* Subcutaneous Route Elicited High Titers of Anti-Ent IgY in Both Serum and Egg Yolk (Trial #2)

We speculated that failure to increase the titer of Ent-specific IgY in egg yolk in Trial#1 could be addressed by optimizing immunization conditions, such as the choice of the carrier protein (33), layer breed (34), and immunization route (35). Therefore, to test this, in pilot Trial #2, three different carrier proteins (BSA, KLH and recombinant CmeC) were used to prepare Ent conjugates. Each conjugate (BSA-Ent, KLH-Ent, or CmeC-Ent) was subcutaneously injected into Rhode Island Red hens.

**Figure 3:**
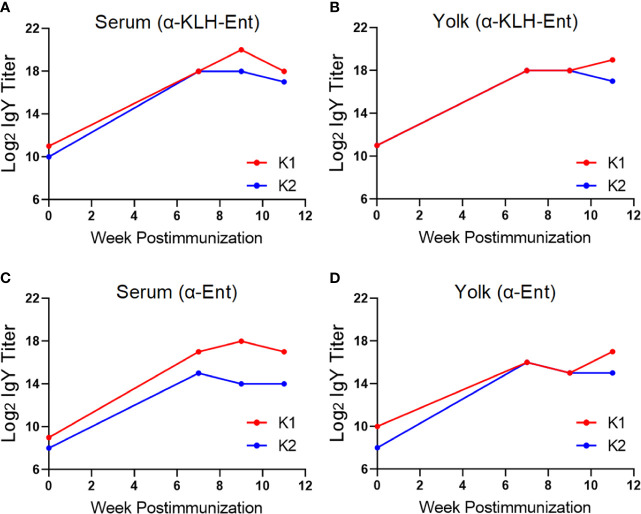
Immune response of Rhode Island Red layers upon subcutaneous immunization with the KLH-Ent conjugate (Trial #2). **(A)** Serum IgY titer against the KLH-Ent conjugate. **(B)** Egg yolk IgY titer against the KLH-Ent conjugate. **(C)** Serum IgY titer against Ent molecule. **(D)** Egg yolk IgY titer against Ent molecule. Each point represents mean IgY titer from duplicate measurements within single chicken. K1 and K2 denote the two individual chickens immunized with KLH-Ent.

With respect to the KLH-Ent conjugate vaccine, immunization triggered strong specific immune response against the whole vaccine at week 11 postimmunization in both serum (128 fold increase for both chickens, **Figure 3A**) and yolk (64 and 256 fold increase in individual chickens, **Figure 3B**) when compared to those in pre-immune samples. Consistently, the titers of the IgY directed against the specific molecule Ent were also increased dramatically in both serum (64 and 256 fold increase, **Figure 3C**) and yolk samples (128 fold increase for both chickens, **Figure 3D**) at week 11 when compared to pre-immune samples.

**Figure 4:**
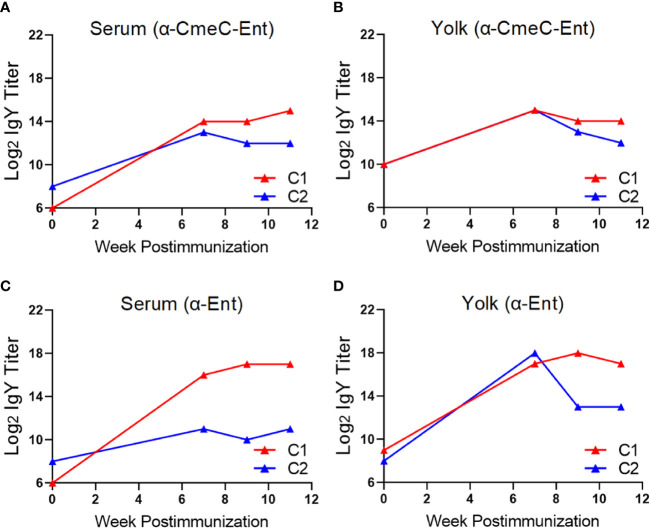
Immune response of Rhode Island Red layers upon subcutaneous immunization with the CmeC-Ent conjugate (Trial #2). **(A)** Serum IgY titer against the CmeC-Ent conjugate. **(B)** Egg yolk IgY titer against the CmeC-Ent conjugate. **(C)** Serum IgY titer against Ent molecule. **(D)** Egg yolk IgY titer against Ent molecule. Each point represents mean IgY titer from duplicate measurements within single chicken. C1 and C2 denote the two individual chickens immunized with CmeC-Ent.

Similarly, for the chickens immunized with CmeC-Ent, significant increase in IgY titer against the whole conjugate was also observed in both serum (16 and 512 fold increase, **Figure 4A**) and yolk (4 and 16 fold increase, **Figure 4B**) at week 11 post immunization. The titers of Ent-specific IgY were also increased dramatically in both serum (8 and 2,048 fold increase, **Figure 4C**) and yolk (32 and 256 fold increase, **Figure 4D**) at week 11 when compared to pre-immune samples. Of note, one chicken (C2 in **Figure 4**) displayed unexpected changes in the titers of anti-Ent IgY in yolk. The level of anti-Ent IgY titers in yolk samples increased dramatically (1,024 fold increase) at 7 week postimmunization but declined to a 32 fold of increase at 9 and 11 week postimmunization when compared to pre-immune samples (**Figure 4D**). The yolk samples from this chicken also displayed fluctuations in IgY titer against whole vaccine, *i.e.*, 32 fold increase at 7 week postimmunization and then 4 fold increase at 11 week postimmunization (**Figure 4B**).

**Figure 5:**
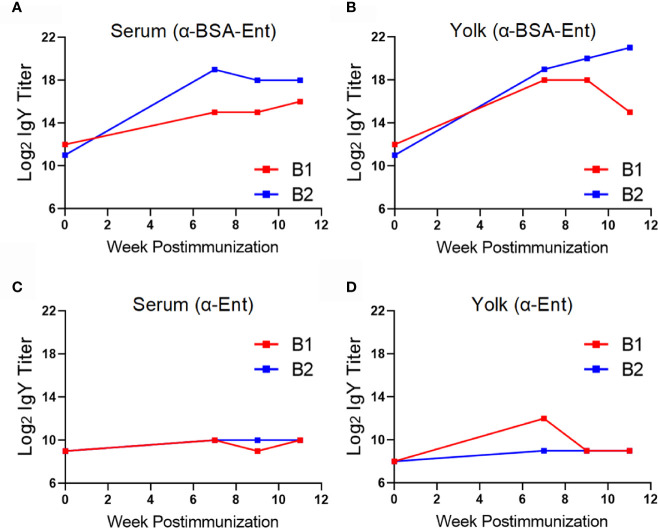
Immune response of Rhode Island Red layers upon subcutaneous immunization with the BSA-Ent conjugate (Trial #2). **(A)** Serum IgY titer against the BSA-Ent conjugate. **(B)** Egg yolk IgY titer against the BSA-Ent conjugate. **(C)** Serum IgY titer against Ent molecule. **(D)** Egg yolk IgY titer against Ent molecule. Each point represents mean IgY titer from duplicate measurements within single chicken. B1 and B2 denote the two individual chickens immunized with BSA-Ent.

Regarding the BSA-Ent conjugate, immunization of chickens could induce high levels of IgY against the whole vaccine (**Figures 5A, B**) but barely elicit Ent-specific IgY in both serum and yolk samples (**Figures 5C, D**). Specifically, BSA-Ent immunization triggered strong specific immune response against the whole vaccine at week 11 in both serum (16 and 128 fold increase, **Figure 5A**) and yolk (8 and 1,024 fold increase, **Figure 5B**) when compared to pre-immune samples. In contrast, Ent-specific IgY titer was only moderately increased in serum (2 fold for both chickens, **Figure 5C**) and yolk (2 fold for both chickens, **Figure 5D**) at week 11 post immunization.

### Subcutaneous KLH-Ent Immunization With Different Vaccination Regimen and Breed Still Triggered High Titers of Anti-Ent IgY in Egg Yolk (Trial #3)

**Figure 6:**
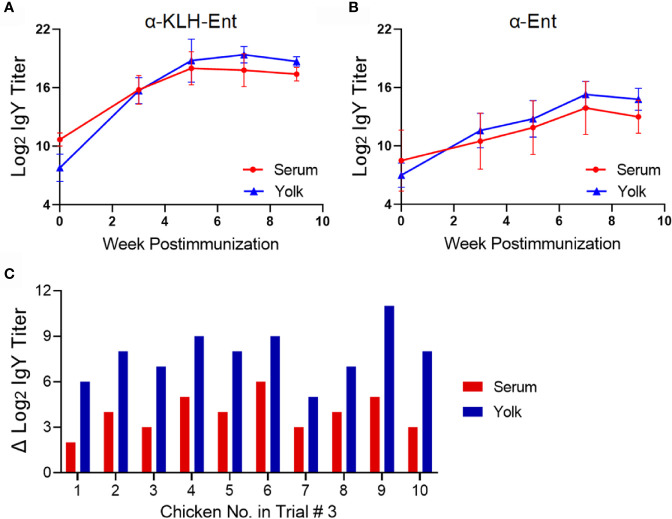
Immune response of White Leghorn layers upon subcutaneous immunization with the KLH-Ent conjugate (Trial #3). A total of ten White Leghorn laying hens received subcutaneous injections with the KLH-Ent conjugate three times (**Figure 1A**). **(A)** Serum and egg yolk IgY titer against the KLH-Ent conjugate. Each point represents mean IgY titer ± standard deviation from ten layers with duplicate measurements within each chicken. **(B)** Serum and egg yolk IgY titer against Ent molecule. Each point represents mean IgY titer ± standard deviation from ten layers with duplicate measurements within each chicken. **(C)** The increase of Ent-specific IgY titer in serum (red bar) and egg yolk (blue bar) in each individual chicken. Y-axis represents the increase in Log_2_ transformed IgY titer from 0 week to 9 weeks postimmunization.

The pilot Trial #2 indicated that subcutaneous immunization of Rhode Island Red pullets with KLH-Ent could significantly induce an Ent-specific immune response. However, this trial was limited by the low number of chickens used in each treatment (n=2). In addition, it was unknown if different choice of breed and vaccination regimen could still lead to the production of high titer of anti-Ent IgY. Thus, a large scale trial (Trial #3) was performed to address these issues, which included using a higher number of chickens for immunization (n=10), using a different industry standard layer breed (White Leghorn), and a reduced number of immunizations (**Figure 1A**, bottom panel). Consistent with the results in Trial #2 (**Figure 3**), the mean IgY titers directed against whole conjugate vaccine dramatically increased 3 weeks after primary immunization, continued to increase upon boost immunizations, and remained at high level at 9 weeks postimmunization in both serum and yolk (**Figure 6A**); notably, this trend was observed in each individual chicken with IgY titer increase in serum ranging from 64 to128 fold and in yolk ranging from 512 to 8,192 fold at 9 weeks postimmunization (data not shown).

The Ent-specific immune response was significantly induced in all White Leghorn chickens (**Figure 6B**), showing similar titer increase pattern as that against whole conjugate (**Figure 6A**). At 9 weeks postimmunization, the mean titer of Ent-specific IgY was increased approximately 16 and 256 fold in serum and egg yolk, respectively (**Figure 6B**). Specifically, the titer of IgY directed against small molecule Ent was drastically increased in each individual chicken with IgY titer increase in serum ranging from 4 to 64 fold and in yolk ranging from 32 to 2,048 fold at 9 weeks postimmunization (**Figure 6C**). Interestingly, the magnitude of anti-Ent IgY increases in egg yolk (up to 2,048 fold) was substantially higher than that in serum (up to 64 fold) in all immunized chickens. **Figure 6C** shows specific increase of anti-Ent IgY titer in serum and yolk samples collected in each of ten individual chickens at 9 weeks postimmunization.

### Ent-Specific Egg Yolk IgY Can Bind Various Ent Derivatives

**Figure 7:**
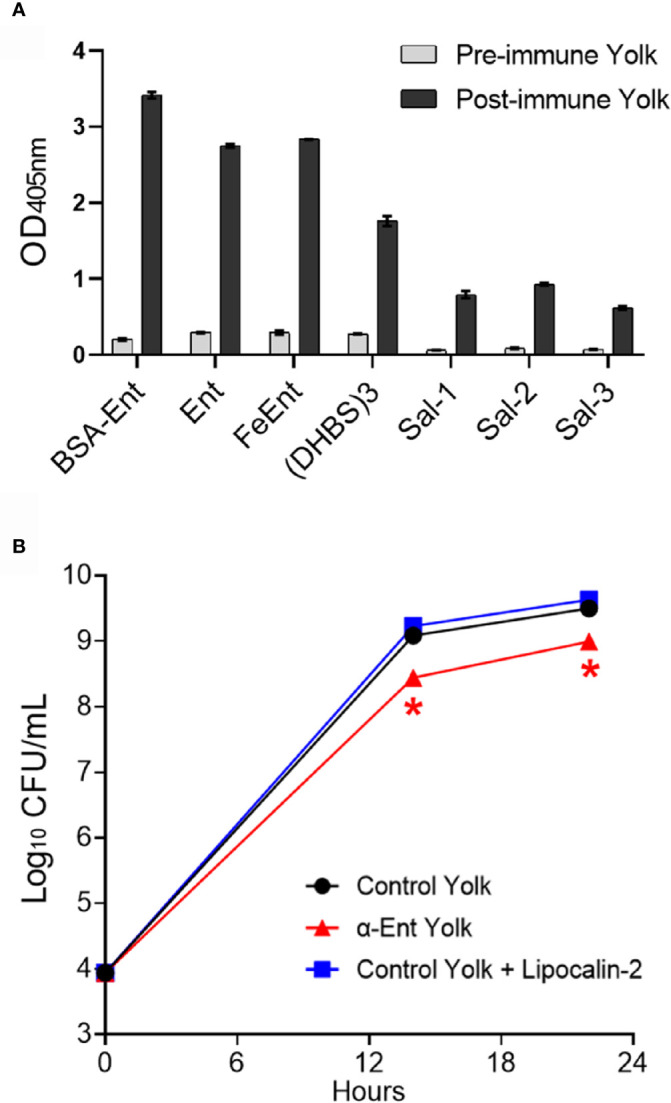
Characterization of anti-Ent egg yolk IgY. **(A)** Binding of anti-Ent egg yolk IgY to different Ent derivatives. Pre-immune and post-immune egg yolk antibodies were diluted by 128-fold and served as the primary antibody in indirect ELISA. BSA-Ent, BSA-Ent conjugate; Ent, *apo* Ent; FeEnt, ferric Ent complex; (DHBS)_3_, linearized Ent; Sal-1, monoglucosyl-Ent; Sal-2, diglucosyl-Ent; Sal-3, triglucosyl-Ent. Error bars represent the standard deviations from at least two independent experiments with duplicate measurements performed in each independent experiment. **(B)** Inhibitory effect of Ent-specific egg yolk antibodies on the growth of *E coli* under iron-restricted condition. The *E coli* MG1655 cells were grown in RPMI medium supplemented with control egg yolk (pre-immune yolk), anti-Ent egg yolk, or the control egg yolk containing human lipocalin-2 (final concentration of 37 µg/mL). At 0, 14 and 22 hours post-inoculation, 20 μL of culture was taken from each well, serially diluted and plated onto LB plates to enumerate CFUs. Each data point represents mean CFU ± standard deviation obtained from duplicate wells in the microtiter plate growth assay. Error bars at different time points are too small to be visualized. Asterisk symbol indicates the significant difference (*P*<0.05) between the control yolk and anti-Ent yolk treatments.

As shown in **Figure 7A**, the post-immune egg yolk IgY displayed significantly increased binding capability to all the tested Ent derivatives including salmochelins (glucosyl Ent derivatives, *i.e.*, Sal-1, Sal-2 and Sal-3) (*P* < 0.05) when compared to pre-immune egg yolk.

### Anti-Ent Egg Yolk IgY Inhibited Growth of *E. coli* MG1655

We also examined growth response of a representative *E. coli* strain (MG1655) to Ent-specific egg yolk IgY using a similar *in vitro* growth assay that was developed for assessment of rabbit Ent antisera (10, 12). As shown in **Figure 7B**, anti-Ent egg yolk displayed significant (*P* < 0.05) inhibitory effect on the growth of *E. coli* MG1655 at 14 and 22 hours after inoculation when compared to the control (**Figure 7B**). Unexpectedly, lipocalin-2 that has served as an ideal positive control to evaluate rabbit anti-Ent serum in previous studies (10, 12) failed to inhibit the growth of MG1655 (**Figure 7B**), suggesting this modified *in vitro* growth assay system may not be optimal for evaluation of the Ent sequestration effect conferred by lipocalin-2.

## Discussion

Iron is a critical element for bacterial metabolism and essential for *in vivo* infections of pathogenic bacteria. Ent-mediated high affinity iron acquisition is a highly conserved and efficient system utilized by enteric bacteria for establishing successful colonization in host (11, 36, 37). In the past decades, several intervention strategies targeting the Ent utilization system have been explored, such as vaccines targeting surface-exposed receptors (38, 39), specific inhibitors blocking Ent biosynthesis (40, 41), and the “Trojan Horse” compounds in which specific antimicrobial was covalently coupled with Ent (42–46). However, these strategies only have limited success, which is likely due to the redundancy, diversity, and/or accessibility of the targeted cellular components.

Recently, a novel immune strategy, which aims to sequester important extracellular siderophores, has gained attention due to the critical role of such highly conserved siderophores, such as Ent, in bacterial pathogenesis. The bacteriostatic effects conferred by host Ent-binding proteins, which include lipocalin-2 (9) and IgA (47, 48), support the feasibility of developing Ent-based immune intervention. Two different types of Ent conjugate vaccines were successfully developed by our laboratory (10) and another independent research group (11). The Ent conjugate vaccine developed by Sassone-Corsi et al. (11) only triggered a moderate Ent-specific IgA response (about 2 fold titer increase) and failed to increase circulating anti-Ent IgG in a mouse model (11). Using a simple and straightforward method, we generated a new type of Ent conjugate vaccine that dramatically induced the level of specific anti-Ent IgG in rabbits (10). More importantly, the rabbit anti-Ent IgG could inhibit *in vitro* growth of diverse strains belonging to three significant Gram-negative pathogens: *E. coli*, *S. enterica*, and *Campylobacter* spp. (10, 12). We also provided compelling evidence that the Ent-specific antibodies induced by the new KLH-Ent conjugate vaccine were superior to host lipocalin-2 in sequestering salmochelins, the modified Ent, consequently inhibiting bacterial growth more effectively (12). Despite exciting findings from the characterization of anti-Ent IgG (10, 12), different titers of Ent-specific antibodies observed in two independent studies (10, 11) suggest that induction of Ent-specific antibodies may be affected by the choice of animal hosts and vaccination regimens. Thus, evaluation of immunogenicity of the new Ent conjugate vaccine (10) in different animal hosts, such as chickens, is highly warranted for developing both active and passive immune intervention strategies against bacterial pathogens.

Recently, passive immunization has regained attention for both animal health and public health, primarily due to increasing antibiotic resistance and a lack of immune response to conventional vaccines in immunodeficient individuals. In case of emergent scenarios, such as treating the patients with developed symptoms, passive immunization through direct administration of specific antibodies may provide immediate protection. Current worldwide trends to limit and even ban antibiotic usage in food animals has raised an urgent need for effective alternatives to antibiotics. The passive immunization through supplementation of hyperimmune egg yolk powder in feed has been recognized as a practical and cost-effective approach to control bacterial pathogens in food animals (15, 16). For example, oral administration of chickens with specific egg yolk antibodies reduced the intestinal load of several important foodborne pathogens including *S.* Typhimurium, *E. coli* O157:H7, and *C. jejuni* and decreased bacterial excretion of 3-4 log_10_ units (49–51). Using mouse model, a recent study (52) showed that clearance rates of *Helicobacter pylori* infection in specific yolk antibody treatment groups were 63.3% higher than sucralfate treatment group and were 21.6% higher than the group receiving the widely adopted dual therapy. Given these promising findings from passive immune intervention using specific egg yolk antibodies, the main goal of this study is to generate high level of Ent-specific egg yolk antibodies using the new Ent conjugate vaccines developed recently (10). Our data demonstrated that subcutaneous immunization of layers with the KLH-Ent conjugate vaccine conjugates could consistently induce high titers of Ent-specific IgY in both serum and egg yolk, which has laid a solid foundation for us to evaluate prophylactic and therapeutic efficacy of the Ent-specific egg yolk antibodies for mitigation of Gram-negative infections in food animals, such as collibacillosis in the poultry industry.

Our results here suggest that choice of immunization route and carrier protein is important for eliciting an Ent-specific immune response in chickens. Compared to the intramuscular injection that failed to induce Ent-specific IgY in egg yolk (**Figure 2D**), subcutaneous immunization of different breeds of layers with the same KLH-Ent vaccine consistently induced a high level of anti-Ent IgY in egg yolk in two independent experiments (**Figures 3D**, **6B**). Subcutaneous injection to the sites close to chicken’s neck in Trial #2 & #3 would make vaccine easily accessible to lymph nodes, consequently eliciting strong immune responses. However, intramuscular injection into pectoral muscle in Trial #1 may not effectively expose vaccine to lymph system for induction of desired level of immune response. BSA appears not to be an appropriate carrier protein to facilitate a chicken host to mount sufficient Ent-specific immune response (**Figures 5C, D**). In contrast, the conjugate using KLH or CmeC as a carrier could trigger a strong Ent-specific immune response in chickens. It is important to mention that CmeC is an attractive subunit vaccine candidate for *Campylobacter* control in poultry because this conserved outer membrane protein plays a critical role in *in vivo* colonization and multidrug resistance of *C. jejuni* (53). Therefore, the CmeC-Ent conjugate tested in this study is also a promising divalent vaccine candidate to reduce *Campylobacter* loads in chickens. Based on the straightforward principle of Ent conjugation developed by our group (10), Ent is expected to be conjugated to diverse carrier proteins that have exposed Lys residues, such as membrane protein-based and/or toxins-based subunit vaccine candidates, consequently generating innovative multivalent vaccines for pathogen control (54).

Similar to the feature of rabbit Ent-specific antibodies (10), the anti-Ent egg yolk IgY also showed high affinity to a panel of diverse Ent derivatives, including salmochelins, the C-glucosylated Ent (**Figure 7A**). Many pathogenic bacteria (such as *E. coli* and *Salmonella* spp.) can synthesize and/or utilize salmochelins (55, 56) to escape the sequestration of iron by lipocalin-2, therefore acquiring growth advantage in inflamed niches in the intestine (57). Recently, we observed that all the tested diverse enteric pathogenic bacteria (22 *E. coli* and 7 *S. enterica* strains) could produce salmochelins (12). However, in this study, anti-Ent egg yolk antibodies displayed much less magnitude of growth inhibition on *E. coli* (**Figure 7B**) than the Ent-specific antiserum examined in our recent studies (10, 12), which is likely due to different *in vitro* growth system used for evaluating egg yolk IgY. In the future, the *in vitro* growth system needs to be optimized and used for investigating a large set of other Gram-negative pathogens. In addition, the *in vitro* growth assay is only the first proof-of-concept step for practical application; thus, evaluation of *in vivo* protective efficacy of anti-Ent egg yolk IgY against Gram-negative pathogens, such as *E. coli*, is highly warranted in the future. Despite these limitations, the findings from *in vitro* characterization of Ent-specific egg yolk antibodies in this study support the feasibility of using Ent-specific egg yolk antibodies for passive immune intervention against several important enteric Gram-negative bacterial pathogens.

Based on the findings from this study, another potential application of the new Ent conjugate vaccine is to confer offspring protection through maternal antibodies in chickens. During hatching, egg yolk antibodies could be translocated into the circulatory system of the embryonic chick and function similarly to naturally produced antibodies (58, 59). Maternal antibodies are much more meaningful for broiler chickens given the quick turnover rate in the broiler production cycle (normally 6-8 weeks). Within such short frame, it is challenging for newly hatched chicks to mount sufficient immune responses upon vaccination against the infections occurring during early stage of development, such as avian pathogenic *E. coli*-caused yolk sac infection, a major cause of mortality of poultry prior to hatch and during the first week post-hatch. Therefore, vaccination of breeder hens with the new Ent conjugate vaccine may not only directly reduce the occurrence of salpingitis and peritonitis in laying birds but also reduce losses due to yolk sac infection through vertical transfer of protective antibodies from the vaccinated hen to her offspring. This hypothesis needs to be examined in the future.

## Data Availability Statement

The original contributions presented in the study are included in the article/**Supplementary Material**. Further inquiries can be directed to the corresponding author.

## Ethics Statement

The animal study was reviewed and approved by Institutional Animal Care and Use Committee at The University of Tennessee and University of Georgia.

## Author Contributions

Conceptualization: XZ, HW, and JL. Methodology: XZ, HW, JL, NB, and CL. Investigation: XZ, HW, CH, CL, NB, and JL. Data analysis: XZ, HW, and JL. Manuscript writing: XZ, HW, and JL. Project administration and funding acquisition: JL, LN, and CL. All authors contributed to the article and approved the submitted version.

## Funding

This study was supported by the NIH grant 1R21AI119462 and by the United States Department of Agriculture National Institute of Food and Agriculture (NIFA) Award No. 2018-67015-28295.

## Conflict of Interest

The authors declare that the research was conducted in the absence of any commercial or financial relationships that could be construed as a potential conflict of interest.
