# Supplementary material for: Evaluation of the Immunogenic Response of a Novel Enterobactin Conjugate Vaccine in Chickens for the Production of Enterobactin-Specific Egg Yolk Antibodies
Source: Front Immunol. 2021 Apr 2;12:629480. doi: 10.3389/fimmu.2021.629480 (PMC8050339; doi:10.3389/fimmu.2021.629480)
Supplement: Supplementary file 1 [file DataSheet_1.pdf]

Supplementary Materials

Evaluation of the immunogenic response of a novel enterobactin conjugate vaccine in chickens for the production of enterobactin-specific egg yolk antibodies

Ximin Zeng[1†], Huiwen Wang[1†], Canghai Huang[1,2], Catherine M. Logue[3], Nicolle L. Barbieri[3], Lisa K. Nolan[3], Jun Lin[1*]

[1]Department of Animal Science, The University of Tennessee, Knoxville, TN 37996, USA;

[2]College of Fisheries, Jimei University, Xiamen, China;

[3]College of Veterinary Medicine, University of Georgia, Athens, GA 30602, USA

Running title: Enterobactin-specific egg yolk antibody

[†]: X.Z. and H.W. contributed equally to this work.

*Corresponding author:

Dr. Jun Lin

Department of Animal Science

The University of Tennessee

2506 River Drive

Knoxville, TN 37996, USA

Phone: (865)974-5598

Fax: (865)974-7297

Email: jlin6@utk.edu

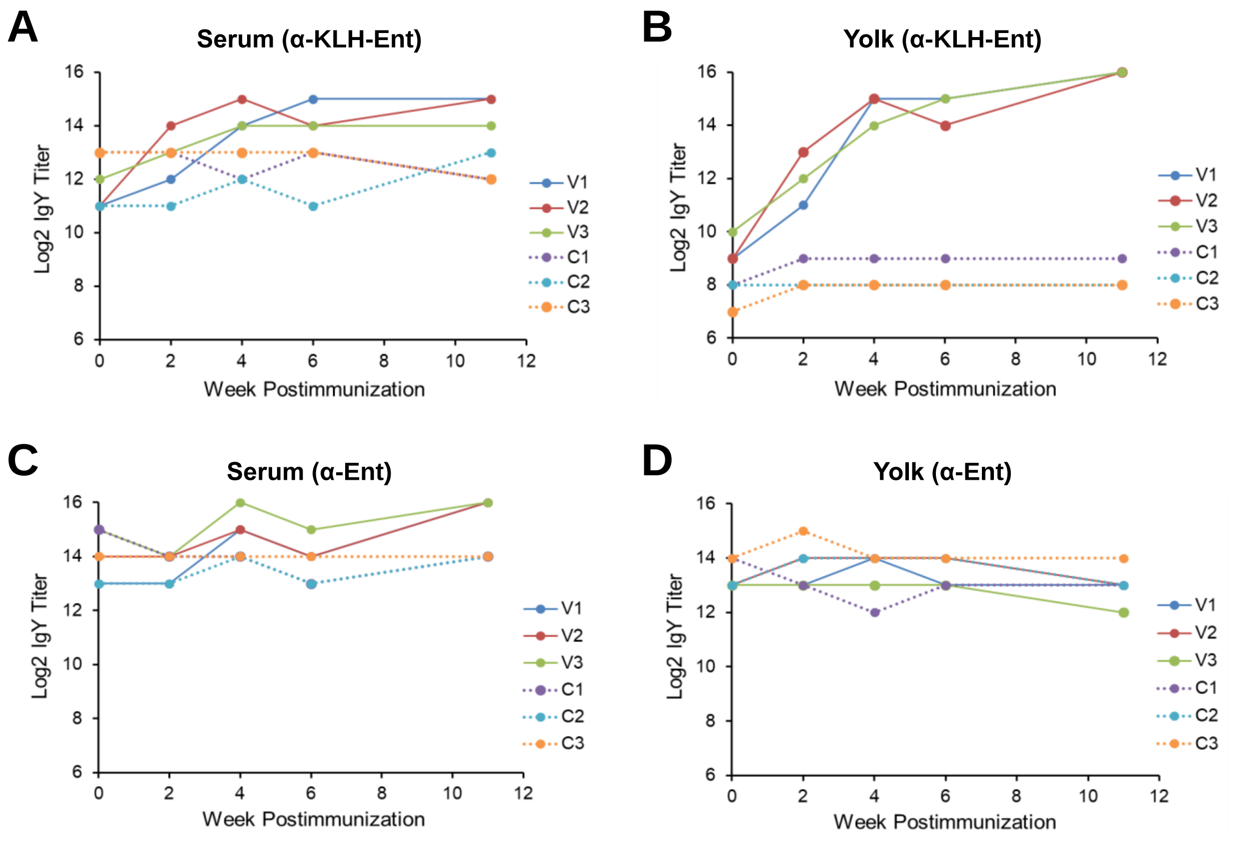

Figure S1. Immune response of Barred Rock layers upon intramuscular immunization with the KLH-Ent conjugate in the individual chickens in control group (C1-C3) and vaccination group (V1-V3) (Trial #1). (A) Serum IgY titer against the KLH-Ent conjugate in each laying hen. (B) Egg yolk IgY titer against the KLH-Ent conjugate in each laying hen. (C) Serum IgY titer against Ent molecule in each laying hen. (D) Egg yolk IgY titer against Ent molecule in each laying hen. Each point represents mean IgY titer from duplicate measurements.
